# Oribatid communities and heavy metal bioaccumulation in selected species associated with lichens in a heavily contaminated habitat

**DOI:** 10.1007/s11356-016-6100-z

**Published:** 2016-01-26

**Authors:** Piotr Skubała, Kaja Rola, Piotr Osyczka

**Affiliations:** Department of Ecology, Faculty of Biology and Environmental Protection, University of Silesia, Bankowa 9, PL-40-007 Katowice, Poland; Department of Plant Taxonomy, Phytogeography and Herbarium, Institute of Botany, Jagiellonian University, Kopernika 27, PL-31-501 Kraków, Poland; Department of Polar Research and Documentation, Institute of Botany, Jagiellonian University, Kopernika 27, PL-31-501 Kraków, Poland

**Keywords:** Oribatid mites, *Cladonia*, Accumulation capacity, Zinc, Lead, Cadmium, Post-smelting wastes

## Abstract

The study examines oribatid communities and heavy metal bioaccumulation in selected species associated with different microhabitats of a post-smelting dump, i.e. three lichen species of *Cladonia* with various growth forms and the slag substrate. The abundance of oribatids collected from the substrate was significantly lower than observed in lichen thalli. The morphology and chemical properties of lichens, and to some extent varying concentrations of heavy metals in thalli, are probably responsible for significant differences in oribatid communities inhabiting different *Cladonia* species. Some oribatids demonstrate the ability to accumulate zinc and cadmium with unusual efficiency, whereas lead is the most effectively regulated element by all species. A positive correlation was found between Zn content in all studied oribatids and their microhabitats. Oribatids exploring different food resources, i.e. fungivorous and non-fungivorous grazers, show considerable differences in bioconcentrations of certain elements.

**Electronic supplementary material:**

The online version of this article (doi:10.1007/s11356-016-6100-z) contains supplementary material, which is available to authorized users.

## Introduction

Smelting activities are of major concern in terms of metallic pollution in industrial regions. The smelting of metal ores, especially using the primitive technology of the past, has resulted in large quantities of wastes deposited as slag dumps, which are entirely artificial. Discarded wastes contain high concentrations of heavy metals, posing a threat to the local environment (Tyszka et al. 2014). The development of vegetation and fauna on heavy-metal-contaminated sites is extremely difficult due to the unfavourable physical and chemical properties of the substrate, as well as the limited pool of species capable of colonising them (Tordoff et al. 2000). Such severe habitat conditions lead to the emergence of ecosystems with a specific species composition.

Lichens (lichenised fungi) are known as stress-tolerators (Grime 1979), and some of them are well-adapted to metal contamination (Purvis and Halls 1996). Therefore, in many heavy-metal-polluted sites, assemblages of lichens constitute the main visual component of vegetation (Cuny et al. 2004; Rola and Osyczka 2014; Rola et al. 2015). The representatives of the lichen genus *Cladonia* are generally known for their morphological dimorphism: first they produce a primary thallus and then variously formed fruticose secondary thalli, called podetia. Due to their fruticose nature and relatively high biomass, some *Cladonia* species appear to be essential and effective rapid colonisers of bare substrates, including extremely contaminated slag dumps (Osyczka and Rola 2013a; Rola et al. 2014).

Many oribatid mites use lichens as a shelter and/or for feeding purposes. Some of them are examples of specialised lichen feeders (Erdmann et al. 2007; Fischer et al. 2010; Seyd and Seaward 1984). Oribatid mites are listed among those animals which exert a grazing impact on lichens (Seyd and Seaward 1984). However, Gjelstrup and Søchting (1979) observed that a lichen, as a whole, apparently does not suffer from the presence of the mite to any appreciable extent. In general, mites are engaged in mutual symbiosis involving all three organisms in the association, i.e. alga, fungus and mite (Gjelstrup and Søchting 1979). Seyd and Seaward (1984) found 83 oribatids in lichens and proposed a detailed classification system based on affinity. Lichens produce many specific secondary metabolites (Huneck and Yoshimura 1996). Seyd and Seaward (1984) reported that no particular lichen substances were repellent to oribatids. However, the toxic relevance of lichen secondary compounds to invertebrates remains controversial. Many studies have shown a defensive role for lichen substances, which may help to protect thalli against herbivores (e.g. Asplund and Gauslaa 2007, 2008; Gauslaa 2005; Pöykkö et al. 2005).

Due to high levels of contamination, the development rate of oribatid fauna is retarded in contaminated dumps (Skubała et al. 2014). Among soil mites, oribatids in particular are able to accumulate metals to very high internal concentrations (Roth 1993; van Straalen and van Wensem 1986). More is becoming known about heavy-metal tolerance, storage and elimination in oribatid mites. For example, the precipitation of metals as intracellular electron-dense granules (EDGs) has been observed in the digestive epithelia of oribatid mites (Alberti et al. 2003; Ludwig et al. 1992). Since some oribatid mites, as edaphic animals, feed on ‘metal-enriched’ fungal mycelia (Roth 1992), they are particularly suited for study of the concentrations of heavy metals.

Although oribatid communities on lichens have been studied by several authors (e.g. Behan-Pelletier and Walter 2000; Erdmann et al. 2006; Fischer et al. 2010; Materna 2000; Seyd and Seaward 1984), the knowledge about these associations in highly contaminated habitats is limited (see Skubała et al. 2014). To recognise the role of lichens in establishing oribatid mite communities, we compared the oribatid fauna associated with three *Cladonia* lichen species and the corresponding substrate derived from slag dumps. Furthermore, to assess the pattern of heavy metal accumulation in the body of oribatids dwelling in different microhabitats (*Cladonia* thalli and dump’s substrate), we analysed metal body burdens in selected oribatid species. More specifically, we addressed the following hypotheses:

- Lichen thalli constitute a less contaminated microhabitat and therefore are populated by richer oribatid fauna than that found on the surrounding slag substrate.
- Oribatid fauna does not differ significantly among various *Cladonia* species.
- The burdens of zinc, lead and cadmium in the body of oribatid mites depend on the contents of these metals in their microhabitats.
- Nutritional metals are accumulated differently than xenobiotics in the bodies of oribatid species.
- Oribatid species feeding mainly on fungi are characterised by higher body burdens of heavy metals.

## Materials and Methods

### Study area and microhabitat description

The study was performed on a post-smelting dump located in the town of Piekary Śląskie (centre of the dump: 55°21′11″N, 18°58′00″E; c. 255,000 m^2^) within the Upper Silesian Industrial Region. The slag dump was deposited as a result of the processing of Zn-Pb ores and consists entirely of artificial slag wastes, which have weathered into a form of friable substrate and partially moulding sinters (Tyszka et al. 2014). Thirty years have elapsed since tipping ceased on this site which is still characterised by unfavourable habitat conditions (Skubała 2011). The average content of organic carbon is low (1.47 %), as is that of total nitrogen (0.08 %); the same is true of the concentrations of macronutrient elements (see dump D2: Osyczka and Rola 2013a). The vegetation on most of the slag dump is characterised by a large proportion of cryptogams, with *Cladonia* spp. clearly predominating (Osyczka and Rola 2013a).

Lichens, and the slag substrate under lichen vegetation, were considered as microhabitats for oribatid mites in this study. Three species of *Cladonia*, representing different growth forms of podetia and producing different types of propagules, were selected: *Cladonia cariosa* (Ach.) Spreng., *Cladonia pyxidata* (L.) Hoffm. and *Cladonia rei* Schaer. (Fig. S1). All three are effective colonisers of post-smelting dumps (Osyczka and Rola 2013a; Rola et al. 2014). *Cladonia cariosa* has torn and fissured podetia, often sparingly branched above, with areolate surfaces and/or surfaces covered with numerous corticated granules. *Cladonia pyxidata* forms typically cup-shaped podetia; the interiors and exteriors of the cups are covered with coarse, corticated granules and/or sparse squamules. The last-mentioned species is generally characterised by long and contorted podetia with partially corticated surfaces densely covered by farinose to granular soredia and squamules which develop to various degrees. Some individuals of *Cladonia rei* are referred to a specific ‘robust/squat’ morphotype of this species described from slag dumps (Osyczka et al. 2014).

### Field studies and sampling

A study plot representing a single stage of succession with a homogenous patch of cryptogamic vegetation was established on the dump. Ten samples of *Cladonia cariosa*, *Cladonia pyxidata* and *Cladonia rei* were randomly collected along with the corresponding substrate to a depth of 5 cm. The lichens (10–20 g) were carefully detached from the surface and packed into zip-lock polythene bags. Substrate samples (40–60 g) were collected using a stainless steel corer. Sampling was done in 2012 during a dry spring season under similar conditions (between 9 and 11 a.m. in sunny weather).

Animals were extracted from samples (lichens and substrate) by means of a Berlese-Tullgren apparatus. Adult individuals and juveniles of *Tectocepheus velatus* were identified after Weigmann (2006); nomenclature follows Subias (2004). Lichen specimens were determined using stereoscopic and light microscopes; chemical analyses of lichen secondary substances were performed by means of thin-layer chromatography (TLC), following Orange et al. (2001). Lichen and substrate samples were dried and weighed, and numbers of mites per 100 g dry weight (DW) were calculated.

### Analysis of substrate and lichen samples

Three samples each of *Cladonia cariosa*, *Cladonia pyxidata* and *Cladonia rei* thalli and three samples of associated substrate were designated for chemical analyses. Macroscopic foreign materials adhering to the thalli surfaces were removed; samples were then rinsed with deionised water, dried at 90 °C for c. 24 h to a constant weight and ground into powder. Concentrations of Zn, Pb and Cd were determined by means of atomic absorption spectrometry. All details concerning sample preparation and chemical analysis are described in Skubała et al. (2014).

### Test species and analytical methods

Five species, i.e. *Ceratozetes mediocris*, *Liochthonius lapponicus*, *Oppiella nova nova*, *Oribatula tibialis tibialis*, *Tectocepheus velatus velatus*, and juveniles of *T. velatus* were selected to study Zn, Pb and Cd concentrations in their bodies and to investigate trends in accumulations of metals in oribatids collected from different *Cladonia* species and the dump’s substrate. A group of 50 specimens were pooled and weighed three times to establish their weight accurately. Other details concerning analysis of metals in the bodies of oribatid species are described in Skubała et al. (2014).

The bioconcentration factor (BCF) was calculated according to the formula: concentration of the metal in the organism/concentration of the metal in the microhabitat. BCF is a parameter used to describe the transfer of trace elements from the soil to oribatid bodies. We used the term ‘accumulator’ for species with a BCF ≥ 1, and ‘non-accumulator’ or ‘deconcentrator’ for oribatids characterised by a BCF < 1.

### Statistical analysis

Four univariate measures were used to assess oribatid community structure: abundance per 100 g DW, total and mean number of species, and the Shannon index (log_2_). One-way analysis of variance (ANOVA) was performed, followed by Tukey’s HSD test, to reveal significant differences in oribatid fauna between the four microhabitats under consideration (three *Cladonia* lichens and the dump’s substrate). Variables deviating from a normal distribution (Kolmogorov-Smirnov test with Lilliefors correction) were transformed using natural logarithms. Significant effects of microhabitat type and oribatid species on particular element concentrations in the bodies of oribatids were calculated by means of multivariate analysis of variance (MANOVA) using Wilks’s lambda test statistic. When the result of MANOVA was significant (*p* < 0.05), univariate tests for each element and Tukey’s HSD post-hoc tests were performed to detect significant differences between particular species and microhabitat types. Additionally, differences between heavy-metal contents in juvenile and adult forms of *T. velatus* were verified by Student’s *t* test.

The interrelationships between concentrations of heavy metals in the bodies of selected oribatid species and content of metals in the corresponding thalli of particular *Cladonia* species and the dump’s substrate were checked using the Pearson correlation coefficient.

A DCA was carried out to determine if the species matrix exhibited a linear or a unimodal response. According to ter Braak and Šmilauer (2002), if the length of the gradient for the first axis is less than 3, the data display a linear response. As the length of gradient equalled 1.057, principal component analysis (PCA) was chosen for the ordination analysis and was applied to the log (*x* + 1)-transformed original abundance data to test for differences in oribatid community structure between microhabitats. Singletons were removed from the analysis.

Statistical calculations were performed using STATISTICA 10 and CANOCO v 4.5 for Windows.

## Results

### Microhabitat conditions

**Fig. 1:**
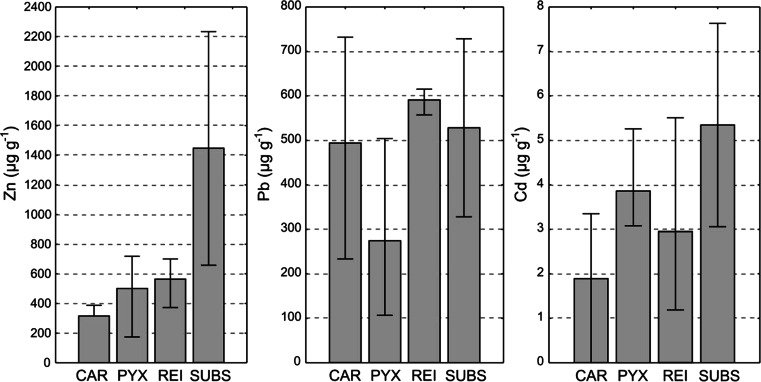
Concentrations of particular metal elements across all microhabitats (*n* = 3). Bars indicate mean values; whiskers show minimum and maximum values. Abbreviations as follow: *CAR*—*Cladonia cariosa*, *PYX*—*Cladonia pyxidata*, *REI*—*Cladonia rei*, *SUBS*—dump’s substrate

As regards the dump’s substrate, the amounts of Zn, Pb and Cd exceeded maximum concentrations for uncontaminated soils by factors of 14, 10 and 5, respectively (cf. Kabata-Pendias and Pendias 2001 and Fig. 1). Amounts of heavy metals in *Cladonia* thalli were lower than in the dump’s substrate. Only the content of Pb in *Cladonia rei* was slightly higher than in the substrate. Generally, different *Cladonia* species accumulated heavy metals at similar levels. However, *Cladonia rei* seems to be a somewhat stronger accumulator of Zn and Pb, whereas *Cladonia pyxidata* absorbed slightly more Cd (Fig. 1).

### Abundance of oribatid mites across microhabitats

In total, 6081 mesofauna specimens were collected, of which 5140 individuals belonged to the Acari taxon, with a significant proportion of oribatid mites (3014 specimens). Most oribatids (59 %) were collected from lichen thalli. The abundance of oribatids collected from the dump’s substrate (5.7 individuals 100 g^−1^ DW) was much lower than in lichen thalli. Among the examined lichen thalli, *Cladonia pyxidata* contained the highest average number of oribatid mites (220.7 indiv. 100 g^−1^ DW) and *Cladonia cariosa* the lowest (25.0 indiv. 100 g^−1^ DW). The number of adult specimens was two (*Cladonia pyxidata*) to four times (*Cladonia cariosa*) higher than juveniles (Table 1). ANOVA revealed significant differences in the abundance of juvenile and adult oribatids among studied microhabitats (Table 1). The proportion of Oribatida relative to the total number of Acari was highest in *Cladonia rei* thalli (71.4 %), while maintaining similar levels in *Cladonia pyxidata* (52.2 %), *Cladonia cariosa* (50 %) and the dump’s substrate (59.2 %).

**Table 1:** Diversity of oribatid mites collected from *Cladonia* species and the associated substrate at the post-smelting dump: abundance (indiv. 100 g^−1^ DW ± S.E.), species richness (mean ± S.E.), Shannon diversity (*H*′) and ANOVA results (*p* = 0.05) for the hypothesis of no effect of microhabitat. Values with identical letters within the same row are not significantly different to each other at the *p* < 0.05 level according to the Tukey’s HSD test. The highest values of selected characteristics are given in italics

	Microhabitats				ANOVA	
Diversity parameters	*Cladonia cariosa*	*Cladonia pyxidata*	*Cladonia rei*	Substrate	*F*	*p*
Oribatida adults	20.5 ± 6.6ab	*152.1 ± 43.9c*	72.4 ± 12.1b	3.1 ± 0.6a	17.714	0.000
Oribatida juveniles	4.5 ± 1.5a	*68.6 ± 19.3b*	23.4 ± 4.4a	2.7 ± 0.6a	18.054	0.000
Oribatida total	25.0 ± 7.9a	*220.7 ± 62.2b*	95.8 ± 16.0a	5.7 ± 1.1a	18.384	0.000
Total number of species	8	16	*38*	21	–	–
Mean number of species	3.5 ± 0.4a	7.0 ± 0.7ab	*10.2 ± 1.7b*	4.8 ± 0.8a	6.894	0.000
H’	0.487 ± 0.1a	1.381 ± 0.1b	*1.400 ± 0.2b*	0.887 ± 0.1ab	5.445	0.002

### Species richness patterns and species composition

Forty-five species of oribatid mites belonging to 31 genera were identified (see Appendix). Differences in species richness between microhabitats were also pronounced. The highest number of species was recorded in *Cladonia rei* thalli (38). The number of oribatid species associated with *Cladonia pyxidata* thalli (16) and *Cladonia cariosa* thalli (8) was much lower, whereas 21 species were recorded in the dump’s substrate. Similarly, the mean number of species was the highest on *Cladonia rei* thalli (10.2) and the lowest on *Cladonia cariosa* thalli (3.5). Significant differences in species richness between particular microhabitats were observed. The Shannon index, generally low for all microhabitats, was the highest in *Cladonia rei* thalli and the lowest in *Cladonia cariosa* thalli (Table 1).

As many as 26 species (58 % of the total number of species) were restricted to one microhabitat (see Appendix). Twenty species occurred exclusively in *Cladonia rei* thalli; however, only *L. propinquus* constituted a substantial part of the mite fauna (12.5 %). Five species were specific to the dump’s substrate, with *Punctoribates punctum* being the most numerous (6.3 %). Six species were common to all microhabitats. *T. velatus* reached a disproportionally high percentage in the total abundance of oribatids (over 50 %) over all studied microsites.

**Fig. 2:**
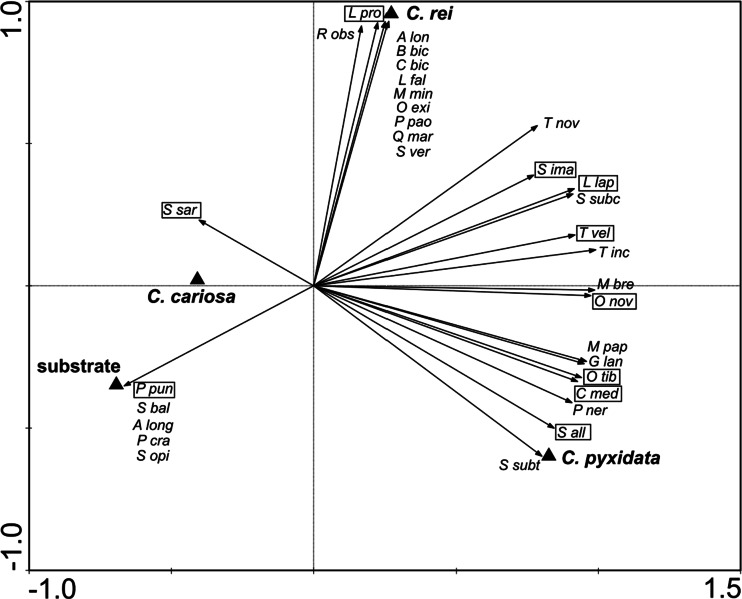
The biplot of the first two axes of the principal component analysis (PCA) of the four microhabitats at the post-smelting dump. Species that constituted more than 2.1 % of the total number are presented in a frame. Abbreviations of the species, see Appendix

The PCA ordination diagram distinguished four different groups of oribatids (Fig. 2). The first axis (72.1 % of the total variance) distinguished between oribatid fauna characteristic of *Cladonia pyxidata* and *Cladonia rei* thalli (right part of the diagram) and other microhabitats (left part). The second axis (21.5 % of the total variance) separated *Cladonia rei* thalli (upper part of axis 2) from *Cladonia pyxidata* thalli (lower part of axis 2). At least three oribatid species can be considered as being associated with *Cladonia pyxidata* thalli, namely *Ceratozetes mediocris*, *Oribatula tibialis* and *Suctobelbella* (*Flagrosuctobelba*) *alloenasuta*. The highest number of species was recorded in *Cladonia rei* thalli; however, only *L. propinquus* was strongly associated with this microhabitat. *Suctobelbella acutidens sarekensis* was typical for *Cladonia cariosa* thalli, whereas *P. punctum* was unique for the dump’s substrate.

### Heavy-metal burdens in oribatid species

Oribatid species have different accumulation capacities for certain heavy metals (Table 2). MANOVA revealed highly significant differences in concentrations of metal elements in the bodies of oribatids with regard to oribatid species and microhabitat type (Wilks’s lambda = 0.003 and 0.105, respectively). The highest concentrations of Zn were found in *Ceratozetes mediocris*, *T. velatus* and *Oribatula tibialis*. The concentrations of Zn in the smallest species among those studied (*L. lapponicus* and *Oppiella nova*) were considerably lower. Even though the concentrations of Zn in juvenile forms of *T. velatus* varied greatly, adult individuals accumulated significantly higher amounts of Zn than juveniles (*t* = 6.90; *p* < 0.001). In general, the amount of Zn in most oribatid species (except *Oribatula tibialis*) collected from the substrate was significantly higher than in individuals collected from lichens (Table 2). As regards Pb, the highest average concentrations were found in *Ceratozetes mediocris*, *Oppiella nova* and juvenile forms of *T. velatus*, whereas the lowest were found in *L. lapponicus* and *Oribatula tibialis*. The best accumulator of Cd was *Ceratozetes mediocris*; the concentration of the metal in this species was four to ten times higher than in other species. *Oppiella nova* and *Oribatula tibialis* were characterised by the lowest concentrations of Cd. In general, the levels of Cd and Pb recorded in oribatids collected from the substrate were higher than in individuals collected from lichens (Table 2).

**Table 2:** Heavy metal concentrations (μg/g of fresh weight; mean ± S.E., *n* = 3) in the oribatids’ bodies and mean bioconcentration factors (BCF, in parentheses) of oribatid species collected from three *Cladonia* species and the substrate on the post-smelting dump. The highest concentrations of particular heavy metals and BCF ≥ 1.0 are given in italics. Mean values with identical letters within the same row are not significantly different to each other at the *p* < 0.05 level according to the Tukey’s HSD test

Element	Microhabitat	Oribatid species					
		*Ceratozetes mediocris*	*Liochthonius lapponicus*	*Oppiella nova*	*Oribatula tibilias*	*Tectocepheus velatus*	*Tectocepheus velatus* (juv.)
	CAR	*1120.0 ± 111.7b (3.5)*	3.2 ± 0.4a (0.01)	35.5 ± 1.8a (0.1)	260.0 ± 29.8a (0.8)	330.0 ± 17.4a *(1.1)*	2.1 ± 0.1a (0.01)
Zn	PYX	*1724.7 ± 512.4b (3.4)*	23.5 ± 3.1a (0.05)	2.0 ± 0.5a (0.004)	426.4 ± 27.3a (0.8)	695.6 ± 74.8a *(1.4)*	260.2 ± 72.9a (0.5)
	REI	*772.6 ± 220.6b (1.4)*	21.5 ± 1.6ab (0.04)	58.6 ± 12.2ab (0.1)	197.2 ± 17.7ab (0.3)	432.5 ± 22.7ab (0.8)	2.0 ± 0.5a (0.004)
	SUBS	*1774.9 ± 298.2b (1.2)*	33.5 ± 3.2a (0.02)	476.0 ± 96.1a (0.3)	370.0 ± 121.6a (0.2)	1520.7 ± 87.4b *(1.0)*	582.0 ± 6.6a (0.4)
Pb	CAR	*75.8 ± 3.8b (0.1)*	68.4 ± 4.8ab (0.1)	34.6 ± 1.4a (0.07)	28.2 ± 2.3a (0.06)	56.1 ± 3.6ab (0.1)	*88.3 ± 6.1b* (0.2)
	PYX	110.8 ± 9.7c (0.4)	12.6 ± 0.7a (0.04)	*131.6 ± 4.8c* (0.05)	44.4 ± 3.2ab (0.2)	58.7 ± 4.8b (0.2)	14.5 ± 0.8a (0.05)
	REI	*81.9 ± 6.1c* (0.1)	34.6 ± 2.9ab (0.06)	24.7 ± 2.2ab (0.04)	6.6 ± 0.8a (0.01)	53.8 ± 14.9bc (0.09)	38.3 ± 1.6ab (0.06)
	SUBS	*178.0 ± 14.6c* (0.3)	29.8 ± 3.0a (0.06)	127.8 ± 12.5b (0.2)	11.6 ± 1.7a (0.02)	168.2 ± 21.0bc (0.3)	145.8 ± 3.1bc (0.3)
Cd	CAR	*8.35 ± 0.5b (4.4)*	0.77 ± 0.8a (0.4)	0.60 ± 0.03a (0.3)	0.72 ± 0.04a (0.4)	0.82 ± 0.07a (0.4)	1.71 ± 0.2a (0.9)
	PYX	*10.32 ± 0.5b (2.7)*	0.85 ± 0.04a (0.2)	0.83 ± 0.08a (0.2)	0.93 ± 0.1a (0.2)	1.32 ± 0.1a (0.3)	0.58 ± 0.06a (0.1)
	REI	*4.37 ± 0.3b (1.5)*	2.18 ± 0.4a (0.7)	0.75 ± 0.1a (0.2)	0.86 ± 0.1a (0.3)	0.90 ± 0.1a (0.3)	0.88 ± 0.1a (0.3)
	SUBS	*8.47 ± 0.8c (1.6)*	3.14 ± 1.0b (0.6)	0.73 ± 0.04a (0.1)	0.89 ± 0.01a (0.2)	2.54 ± 0.2ab (0.5)	1.88 ± 0.04ab (0.3)

Microhabitats: *CAR Cladonia cariosa*, *PYX Cladonia pyxidata*, *REI Cladonia rei*, *SUBS* substrate

The calculated values of BCF are presented in Table 2. The noticeable Zn enrichment was found for two oribatid mites, *Ceratozetes mediocris* and adult forms of *T. velatus* (Table 2). Although lichen thalli and the dump’s substrate were seriously contaminated with Pb, oribatid species were not enriched with the metal, as the concentrations of Pb in the bodies of oribatids were much lower relative to those recorded in their microhabitats. *Ceratozetes mediocris* also appears to be an accumulator of Cd, with BCF varying from 1.5 in *Cladonia rei* to 4.4 in *Cladonia cariosa*. In contrast, other oribatid species can be described as deconcentrators of this metal; however, the value of BCF for all species was not as low as in the case of Pb. The bioconcentration factor differs slightly between studied microhabitats; however, it was not possible to find a general rule (Table 2).

A significant positive correlation was found between the contents of Zn in the bodies of oribatid species and corresponding microhabitats in the case of all studied species. A negative or very low positive correlation was recorded for Cd. As regards Pb, negative correlations (*Oribatula tibialis*, *Oppiella nova*) or slightly positive correlations (other species) were detected (Table 3).

**Table 3:** Pearson’s correlation coefficients (*R*) between the content of heavy metals in the bodies of selected oribatid species vs. content of metals in microhabitats (lichen thalli and substrate) at the post-smelting dump

Oribatid species	Zn	Pb	Cd
*Ceratozetes mediocris*	0.63*	0.05	0.24
*Liochthonius lapponicus*	0.65*	0.21	0.17
*Oppiella nova*	0.90*	−0.33	0.22
*Oribatula tibialis*	0.62*	−0.59*	−0.04
*Tectocepheus velatus*	0.69*	0.14	0.37
*Tectocepheus velatus* (juv.)	0.64*	0.31	−0.04

*Significant at *p* < 0.05

## Discussion

### Zoocoenotic characteristics of oribatid fauna in *Cladonia* species and the dump’s substrate

The number of species recorded on lichens was twice that in the dump’s substrate and oribatid mite abundance was 4 times (*Cladonia cariosa*) to over 35 times (*Cladonia rei*) higher on thalli than in the corresponding substrate. The comparatively high number of oribatid species in the thallus of lichens is probably due to invasion by soil-inhabiting oribatids. This indicates that lichens constitute a specific habitat which, isolated from the direct influences of the surrounding substrate, seems more suitable for oribatid mites.

A clear preference for lichen thalli over the surrounding substrate by oribatid mites at the dump is similar to a phenomenon observed in corticolous arthropods on tree trunks. The minute structures of lichen thalli positively influence the abundance and living conditions of various arthropods occurring on the bark (Prinzing and Wirtz 1997). Lichen thalli might protect against excessive solar radiation, wind and desiccation. The benefits are mutual, as oribatid mites play an important role in the dispersal of lichen spores and propagules by transporting them on body surfaces or in the gut (Gerson and Seaward 1977; Root et al. 2007; Seyd and Seaward 1984; Stubbs 1995).

*Cladonia* lichens proved to be rather weak accumulators; heavy-metal concentrations in their thalli are considerably lower than in the host substrate (Osyczka and Rola 2013b). Consequently, it can be concluded that oribatid mites choose these lichens not only as a shelter or source of food but also as less-contaminated microhabitats. This phenomenon supports the idea that oribatid mites resist heavy metals by selecting less-contaminated microsites (Bengtsson et al. 1994; Skubała et al. 2014; Tranvik and Eijsackers 1989). Furthermore, since the oribatids are sensitive to disturbances caused by enchytraeids and earthworms, they may take advantage of increased space and resources due to the scarcity of those metal-sensitive organisms (Creamer et al. 2008; Maraun and Scheu 2000; Maraun et al. 2003).

Oribatid communities differed not only between lichens and the dump’s substrate but also among different species of *Cladonia*. A significantly higher abundance of oribatids was recorded on the thalli of *Cladonia pyxidata* in comparison with two other lichens, whereas the highest total and mean number of species were recorded on *Cladonia rei*. The observed differences in the oribatid fauna are probably due to the various morphological structures of lichen thalli. Colloff (1988) remarked that thallus morphology has a definite influence on oribatid species diversity. Both species richness and abundance of oribatids may depend on the growth form of lichens (Fröberg et al. 2003; Root et al. 2007; Smrž and Kocourková 1999). All of the *Cladonia* species considered in this study are fruticose; however, they apparently differ in micromophological structure. *Cladonia rei* demonstrates great variability in the growth forms of its podetia, which are richly covered with various propagules, such as corticated granules, squamules and microsquamules as well as numerous non-corticated, farinose-to-granular soredia (see Dolnik et al. 2010; Osyczka et al. 2014). Therefore, *Cladonia rei* has a greater surface area/volume ratio in comparison to other studied *Cladonia* species. This species provides more microniches for tiny oribatid mites and for gathering of the organic matter particles which constitute their nourishment. In contrast, the morphology of *Cladonia cariosa* and *Cladonia pyxidata* is less diversified; their podetia do not produce soredia (see James 2009). Consequently, the huge morphological variability of *Cladonia rei* could be the main reason it is characterised by the highest diversity of oribatids among examined species. Another important factor affecting oribatid communities on lichens may be associated with the production of secondary metabolites by mycobionts. Some of these compounds have been shown to exhibit activity which is toxic and antifeedant to invertebrates (see Huneck 1999; Molnár and Farkas 2010). The low levels of diversity and abundance of oribatids on the thalli of *Cladonia cariosa* could be connected with the storage of atranorin in its cortex (Reutimann and Scheidegger 1987), which has been shown to be a deterrent to herbivory and to exhibit toxic effects on some invertebrates (Nimis and Skert 2006; Pöykkö et al. 2005; Slansky 1979). *Cladonia pyxidata* produces fumarprotocetraric acid, which also exerts an adverse effect on invertebrates and may help to protect the thalli against herbivores (Hesbacher et al. 1995). However, the abundance of oribatids was much higher on the thalli of *Cladonia pyxidata* than on other species. Fumarprotocetraric acid in *Cladonia pyxidata* is confined exclusively to the medulla and appeared to be either lacking or negligible in the algal and cortical layers. Thus, oribatids feeding on *Cladonia pyxidata* could easily avoid the uptake of this lichen compound from the interior parts of thalli, as found in the feeding preferences of *Eilema complana* larvae (Hesbacher et al. 1995). Individuals of *Cladonia rei* examined in this study contained only homosekikaic acid, with no toxic effect determined to date. This may be an additional attribute of *Cladonia rei* as a suitable microhabitat for various oribatids. Furthermore, the high abundance of oribatids on *Cladonia pyxidata* could also be the result of relatively low concentrations of toxic Pb in the thalli (Fig. 1). However, with regard to Zn and Cd, there was no clear relationship between content of metals and abundance or species richness of oribatids in the lichen thalli.

Some mites strongly prefer certain lichen species (Fröberg et al. 2003). In our study, according to the classification of Seyd and Seaward (1984), none of the recorded oribatids represent group A (species restricted to lichens as a biotope) or B (species preferring lichens as a habitat). Seven species could be classified within group C, which includes oribatids frequently associated with lichens but equally common in other habitats (see Appendix). Nevertheless, the differences in oribatid composition among studied lichens are apparent; some oribatids proved to be strongly linked to particular *Cladonia* species (Fig. 2). Generally, oribatid species dwelling *Cladonia* lichens are exposed to similar source of food. Therefore, microhabitat properties (i.e. morphology of thallus, secondary metabolite production, content of heavy metals), not nutritional factors, are probably responsible for a mite’s preferences (see also Gerson and Seaward 1977; Lawrey 1987).

### Bioaccumulation of heavy metals in oribatid species

High interspecific variation in the concentrations of heavy metals has been observed in our study. Zinc is an essential element required in several key enzymes (Rainbow 1988; Taylor and Simkiss 1984); thus, one might expect that animals are able to regulate its internal concentrations at a more or less fixed level. This ability may differ among species due to varying storage or elimination capacities. Our results fit well with those of other authors who also observed a wide variation in Zn body burden in oribatids (Skubała and Kafel 2004; Skubała and Zaleski 2012; Zaitsev and van Straalen 2001). However, the ability of *Ceratozetes mediocris* to accumulate Zn in high quantities had not been documented so far. The species and adult forms of *T. velatus* appeared to be an accumulator of Zn irrespective of their microhabitats. Furthermore, besides *Oppiella nova* and *Oribatula tibialis* (analysed by Skubała and Kafel 2004 and Skubała and Zaleski 2012), *L. lapponicus* was found to be the deconcentrator of Zn. The Pb accumulation capacity of oribatids is generally low; relatively comparable concentrations of this element were observed in the specimens. It seems that the investigated species are able to prevent high internal Pb concentrations, possibly due to low uptake through the gut wall or by rapid excretion of Pb (Janssen and Hogervorst 1993). Cd concentrations in the oribatid bodies are the lowest and the least variable, except for *Ceratozetes mediocris*, which turned out to be an accumulator of this metal. Other authors did not observed a general rule for Cd accumulation by oribatids (El-Sharabasy and Ibrahim 2010; Janssen and Hogervorst 1993; Skubała and Kafel 2004; Skubała and Zaleski 2012; Zaitsev 1999).

*L. lapponicus*, *Oppiella nova*, *Oribatula tibialis* and juveniles of *T. velatus* can be categorised as non-accumulators of all studied heavy metals. Generally, *Ceratozetes mediocris* showed the highest burdens of Zn, Cd and Pb irrespective of microhabitat (Table 2); thus, it can be clearly regarded as an accumulator of the first two heavy metals. Adults of *T. velatus* tend to accumulate more Zn than is present in their microhabitat. It is evident that *Ceratozetes mediocris* and *T. velatus*, which were abundant at the dump, must be characterised by an innate or evolved (physiological) resistance to heavy metals that enables them to maintain their populations at highly contaminated sites. Nevertheless, it should be borne in mind that concentrations in the bodies of different individuals of the same species can vary considerably depending on the life stage of the individuals, as was observed in the case of Zn in *T. velatus* (Table 2). Time of exposure to heavy metals can affect the amount of a given element accumulated in bodies; however, this is probably not a general rule for oribatids (Skubała and Kafel 2004).

Concentrations of heavy metals are as a rule higher in oribatids collected from the dump’s substrate than those from lichen thalli. The strong correlation between the content of metals in oribatids and the corresponding microhabitats was observed only for Zn (Table 3). This is not surprising, as nutritional metals (e.g. Zn) are regulated differently than xenobiotics (e.g. Pb and Cd) in the bodies of oribatid species. Similarly, Skubała and Zaleski (2012) and Skubała et al. (2014) observed that Zn contents in the bodies of *T. velatus* were significantly correlated with contents in the corresponding substrate. On the contrary, Skubała and Kafel (2004) found internal Zn concentrations to be independent of concentrations in forest soil.

According to Siepel (1995), herbofungivorous and fungivorous grazers demonstrate significantly higher body burdens of heavy metals than other mite species in Pb-contaminated environments. It is widely believed that they are more affected by heavy-metal uptake than other feeding guilds of oribatids because they digest fungi cell walls, where large amounts of metals are accumulated (Mowl and Gadd 1984; Trevors et al. 1986). In our study, species utilising different food resources show considerable differences in bioaccumulation of certain metals. However, contrary to expectations, *L. lapponicus*, *Oppiella nova* and *Oribatula tibialis* (fungivorous grazers) have lower body burdens of heavy metals than *Ceratozetes mediocris* (a panphytophage and herbivore) and *T. velatus* (an opportunistic herbofungivore). Apart from species-specific differences in metal accumulation, the pattern depends also on the element under consideration, as was reported by Skubała and Kafel (2004), and its bioavailability in the environment (Zaitsev and van Straalen 2001).

## Conclusions

- Thalli of *Cladonia* lichens represent a less-contaminated microhabitat in heavily contaminated dumps, provide shelter from desiccation, retain humidity and are therefore populated by more numerous and richer oribatid fauna than the surrounding dump’s substrate.
- Noticeable and significant differences in oribatid fauna among three *Cladonia* species are probably due to the morphological characteristics of thalli, production of different secondary metabolites and to some extent varying concentrations of heavy metals in the thalli of particular species.
- High interspecific variation in the concentrations of heavy metals (especially Zn) is characteristic of oribatid species. Zinc (as nutrient) is accumulated with unusual efficiency in some oribatid species (*Ceratozetes mediocris* and *T. velatus*), irrespective of their microhabitats. Lead and cadmium (xenobiotics) are more efficiently regulated by most oribatid species.
- The putative tendency of fungivorous grazers towards greater accumulation of heavy metals in comparison to non-fungivorous ones is not a general rule.

## Electronic supplementary material

Below is the link to the electronic supplementary material.

Fig. S1 Assemblages of studied lichen species of *Cladonia*. (a) *Cladonia cariosa* (Ach.) Spreng. (b) *Cladonia pyxidata* (L.) Hoffm. (c) *Cladonia rei* Schaer. *Scale* = 2cm. (DOC 1.05 mb)

## Appendix

**Table 4:** Abundance (indiv. 100 g^−1^ DW) of oribatid species recorded on *Cladonia cariosa*, *Cladonia pyxidata*, *Cladonia rei* thalli and the dump’ substrate

Species	Abbr.	*C. cariosa*	*C. pyxidata*	*C. rei*	Substrate
*Acrogalumna longipluma longipluma* (Berlese, 1904)	A long	–	–	–	0.01
*Adoristes* (*A.*) *ovatus ovatus* (Koch, 1839)		–	–	0.11	–
*Autogneta* (*A.*) *longilamellata longilamellata* (Michael, 1885)	A lon	–	–	0.73	0.01
*Berniniella* (*B.*) *bicarinata* (Paoli, 1908)	B bic	–	–	0.34	–
*Berniniella* (*B.*) *conjuncta* (Strenzke, 1951)		–	–	0.10	–
*Ceratozetes* (*C.*) *mediocris* Berlese, 1908	C med	0.13	46.75	3.07	0.63
*Cultroribula bicultrata* (Berlese, 1905)	C bic	–	–	0.64	–
*Dissorhina ornata ornata* (Oudemans, 1900) ^C^		–	–	0.11	–
*Euphthiracarus* (*E.*) *cribrarius cribrarius* (Berlese, 1904)		–	0.22	–	–
*Euphthiracarus* (*E.*) *monodactylus* (Willmann, 1919)		–	–	0.11	–
*Eupelops tardus* (Koch, 1835)		–	–	0.11	–
*Galumna* (*G.*) *lanceata* (Oudemans, 1900)	G lan	–	0.86	0.27	0.04
*Lauroppia falcata falcata* (Paoli, 1908)	L fal	–	–	0.99	–
*Licneremaeus licnophorus* (Michael, 1882)		–	–	0.13	–
*Liochthonius* (*L.*) *lapponicus* (Trägårdh, 1910) ^C^	L lap	2.36	9.31	8.72	0.12
*Liochthonius* (*L.*) *propinquus* Niedbała, 1972	L pro	–	–	9.04	–
*Metabelba* (*M.*) *papillipes* (Nicolet, 1855)	M pap	–	2.03	0.50	0.01
*Metabelba* (*M.*) *pulverulenta* (Koch, 1839)		–	–	0.10	–
*Micreremus brevipes* (Michael, 1888) ^C^	N bre	–	0.22	0.13	–
*Microppia minus minus* (Paoli, 1908)	M min	–	–	0.31	–
*Moritzoppia* (*M.*) *unicarinata unicarinata* (Paoli, 1908)		–	–	0.11	–
*Oppiella* (*O.*) *nova nova* (Oudemans, 1902)	O nov	0.15	5.73	2.35	0.17
*Oribatula* (*O.*) *tibialis tibialis* (Nicolet, 1855) ^C^	O tib	0.13	4.56	0.74	0.05
*Oribatula* (*Zygoribatula*) *exilis exilis* (Nicolet, 1855) ^C^	O exi	–	–	0.23	–
*Pantelozetes paolii* (Oudemans, 1913)	P pao	–	–	0.27	–
*Pergalumna nervosa nervosa* (Berlese, 1914)	P ner	–	1.30	0.22	0.04
*Pilogalumna crassiclava crassiclava* (Berlese, 1914)	P cra	–	–	–	0.02
*Punctoribates* (*P.*) *punctum* (Koch, 1839)	P pun	–	–	–	0.19
*Quadroppia* (*Q.*) *maritalis* Lions, 1982	Q mar	–	–	0.38	0.02
*Ramusella* (*R.*) *clavipectinata* (Michael, 1885)		–	–	0.10	–
*Rhinoppia obsoleta* (Paoli, 1908)	R obs	–	–	0.14	0.02
*Sellnickochthonius immaculatus* (Forsslund, 1942)^C^	S imm	0.81	1.05	1.09	0.03
*Subiasella* (*Lalmoppia*) *quadrimaculata* (Evans, 1952)		–	–	0.13	–
*Suctobelbella* (*Flagrosuctobelba*) *alloenasuta* Moritz, 1971	S all	–	0.54	0.10	0.08
*Suctobelbella* (*Flagrosuctobelba*) *baloghi* (Forsslund, 1958)	S bal	–	–	–	0.02
*Suctobelbella* (*Flagrosuctobelba*) *nasalis* (Forsslund, 1941)		–	–	0.10	–
*Suctobelbella* (*S.*) *acutidens sarekensis* (Forsslund, 1941)	S sar	0.55	–	0.11	0.02
*Suctobelbella* (*S.*) *opistodentata* (Golosova, 1970)	S opi	–	–	–	0.02
*Suctobelbella* (*S.*) *similis* (Forsslund, 1941)		–	–	0.14	–
*Suctobelbella* (*S.*) *subcornigera subcornigera* (Forsslund, 1941)	S sub	–	1.31	1.41	0.04
*Suctobelbella* (*S.*) *subcornigera vera* (Moritz, 1964)	S ver	–	–	0.66	–
*Suctobelbella* (*S.*) *subtrigona* (Oudemans, 1900)	S subt	–	0.56	–	0.01
*Tectocepheus velatus velatus* (Michael, 1880)^C^	T vel	16.18	76.33	36.99	1.57
*Trichoribates* (*T.*) *novus novus* (Sellnick, 1928)	T nov	–	0.66	1.22	–
*Trichoribates* (*Latilamellobates*) *incisellus incisellus* (Kramer, 1897)	T inc	0.15	0.67	0.47	–

C—representative of group C (Seyd and Seaward 1984)
