# Supplementary material for: Oribatid communities and heavy metal bioaccumulation in selected species associated with lichens in a heavily contaminated habitat
Source: Environ Sci Pollut Res Int. 2016 Jan 26;23:8861–71. doi: 10.1007/s11356-016-6100-z (PMC4850176; doi:10.1007/s11356-016-6100-z)

**Fig. S1** Assemblages of studied lichen species of *Cladonia*. (a) *C. cariosa* (Ach.) Spreng. (b) *C. pyxidata* (L.) Hoffm. (c) *C. rei* Schaer. Scale = 2cm.


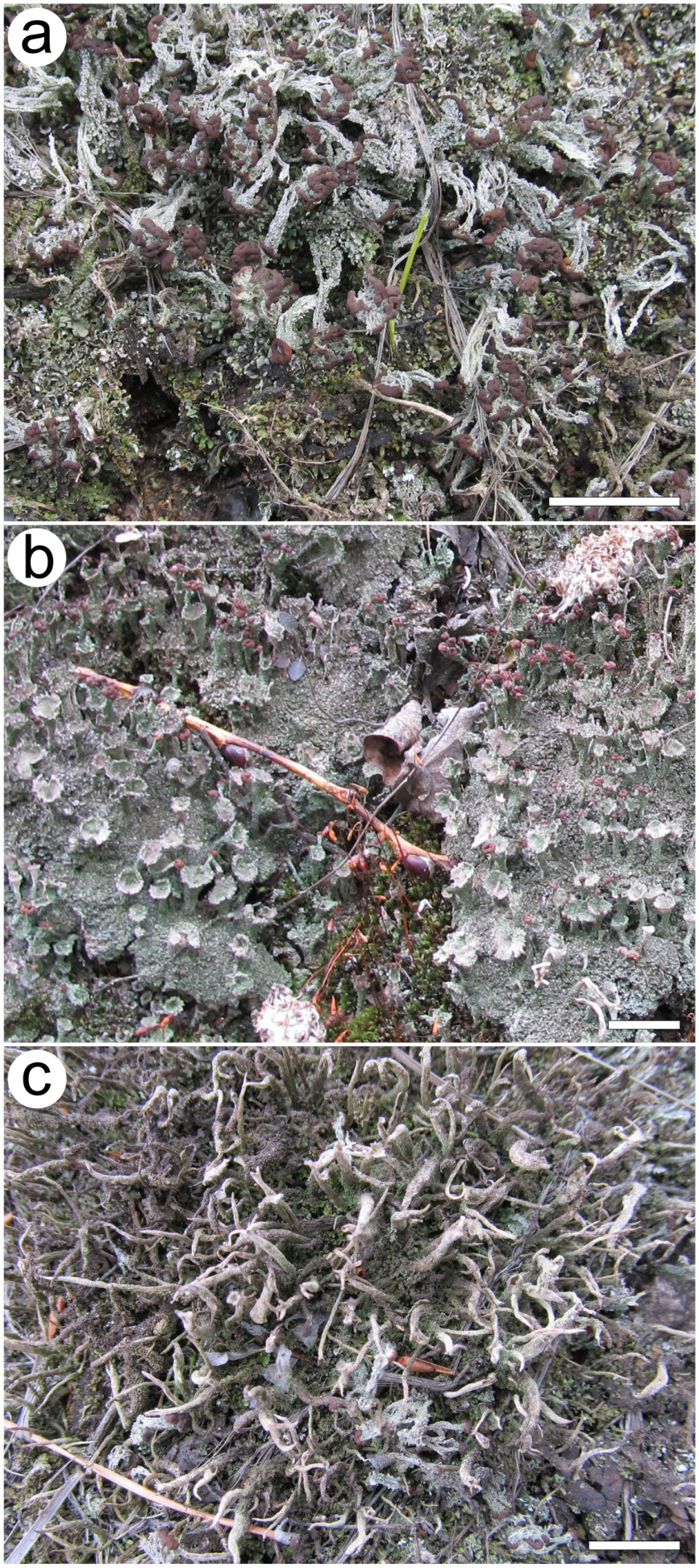

Supplement: Supplementary file 1 — Assemblages of studied lichen species of Cladonia. (a) Cladonia cariosa (Ach.) Spreng. (b) Cladonia pyxidata (L.) Hoffm. (c) Cladonia rei Schaer. Scale = 2cm. (DOC 1.05 mb) [file 11356_2016_6100_MOESM1_ESM.doc]
